# Inequalities in access to community-based diabetes examination and its impact on healthcare utilization among middle-aged and older adults with diabetes in China

**DOI:** 10.3389/fpubh.2022.956883

**Published:** 2022-09-16

**Authors:** Qingwen Deng, Yan Wei, Yingyao Chen

**Affiliations:** National Health Commission Key Laboratory of Health Technology Assessment, School of Public Health, Fudan University, Shanghai, China

**Keywords:** inequality, community-based, diabetes, healthcare utilization, propensity score matching

## Abstract

Globally, diabetes and its complications are becoming one of the leading challenges in health governance. As health inequalities and primary care services related to diabetes are gaining traction, the status of community-based diabetes examination largely remains unclear in the literature. This study aims to investigate inequalities in access to community-based diabetes examination among people with diabetes and to analyze its impact on healthcare utilization. Data from the 2018 China Health and Retirement Longitudinal Study (CHARLS) were applied, and a total of 767 patients with diabetes were included. Inequalities in community-based diabetes examination were illustrated by the concentration curve and normalized concentration index. Propensity score matching (PSM) were used to identify the impact of community-based diabetes examination on outpatient and inpatient care utilization. We found that community-based diabetes examination was accessible to 23.08% of the respondents, of which 76.84% were free, and the highest frequency was 2–6 times per year, accounting for 47.46%. Community-based diabetes examinations were more concentrated among people with poorer-economic condition (95% confidence interval, 95%CI = −0.104, *p* = 0.0035), lower-education level (95%CI = −0.092, *p* = 0.0129), and less-developed areas (95%CI = −0.103, *p* = 0.0007). PSM analyses showed that community-based diabetes examination increased the utilization of outpatient care (odds ratio, OR = 1.989, 95%CI = 1.156–3.974) and decreased the use of inpatient care (OR = 0.544, 95%CI = 0.325–0.909), and the sensitivity analyses confirmed the robustness of the results. This study is the first to examine the status and inequalities of community-based regular diabetes examination and its effect on the likelihood of healthcare utilization among patients with diabetes. The findings suggest that the overall level of community-based diabetes examination is low, and there are pro-socioeconomically disadvantaged inequalities. The value of community-based diabetes examination should be recognized to help person with diabetes face up to their health needs for better disease control and health promotion.

## Introduction

Globally, diabetes and its complications are becoming one of the leading challenges in health governance. As reported by the International Diabetes Federation (IDF), approximately 537 million adults were living with diabetes in 2021 and had caused nearly one trillion dollars in health expenditure (1). According to a recent study conducted by the Chinese Center for Disease Control and Prevention, the overall prevalence of diabetes and prediabetes were estimated to be 12.4 and 38.1% (2). In terms of numbers, China is at the epicenter of the world diabetes epidemic. Worst of all, the rates of awareness, treatment, and adequate control are low and even seem to have stagnated (3, 4). In the face of the heavy disease burden, a major call is to strengthen diabetes self-management and glycemic control *via* regular diabetes examination such as blood glucose monitoring (5–7). Going to the hospital for tests is time-consuming and expensive (such as transportation fees), and performing the tests at home requires the acquisition of equipment and certain knowledge; community-based care services have the advantages of convenience and affordability (8, 9) and are an important aid to achieve a more balanced transition from the hospital to the individual, becoming an increasingly advocated mode of care in China. Routine prevention and management of chronic diseases are critical components of community healthcare provision, and their effective implementation contributes to increased knowledge and ability to control disease, improved health status, and cost savings, as evidenced by several previous studies on diabetes (10–13).

However, the fact is that with limited access to diabetes examinations and a general lack of patient education, a considerable proportion of diabetes cases go undiagnosed or are poorly controlled (2). Previous research has established that one significant factor for this is the absence of primary care actions (14). As a priority population for chronic disease management in the community, regular diabetes examination for people with diabetes is pinned on the hope that it will improve these conditions and facilitate the achievement of health system goals.

Compared to specialized services, primary healthcare services are relatively low cost and sometimes provided free of charge; nevertheless, inequalities in community-level healthcare may still exist even for people living in generous welfare states with universal health coverage (15). It is an undisputed fact that communities of different socioeconomic status (SES) have varying levels of access to health-promoting resources and services (16), particularly in developing countries. While health equity is the focus of attention across countries at the moment, the reality is that the balance is becoming increasingly skewed. Many studies have shown not only health inequalities but also disparities in healthcare utilization among different diabetes populations (17–23). Specifically, a lower SES is associated with a higher prevalence, poorer glycemic control, and more complications (17, 18). Disadvantaged people with diabetes lack the ability to acquire diabetes information (19), lack self-management efforts (20), do not have adequate access to diabetes care (21, 22), and may even avoid healthcare (23). Related issues have aroused great attention in light of the growing demand for healthcare caused by diabetes, but there is little research available on community-based diabetes examination, and our understanding of both its status and actual impact on healthcare utilization is extremely limited. The purpose of this study is to address these two questions.

Andersen's model is the most widely used theoretical foundation for forecasting the factors of healthcare utilization and can serve as a solid conceptual framework for this study. The determinants are categorized into four sets, namely contextual factors, individual characteristics, health behaviors, and health outcome (24), of which the proposed community-based diabetes examination is an important contextual factor. Gaps or inequities in access must be addressed before people with diabetes can benefit from larger community-based diabetes examination to meet health needs and enhance health. Increased understanding is of great value for researchers and policymakers to develop targeted initiatives to promote overall health equity and reasonable healthcare utilization. This study may provide some clues to this end.

## Materials and methods

### Data source and study sample

**Figure 1:**
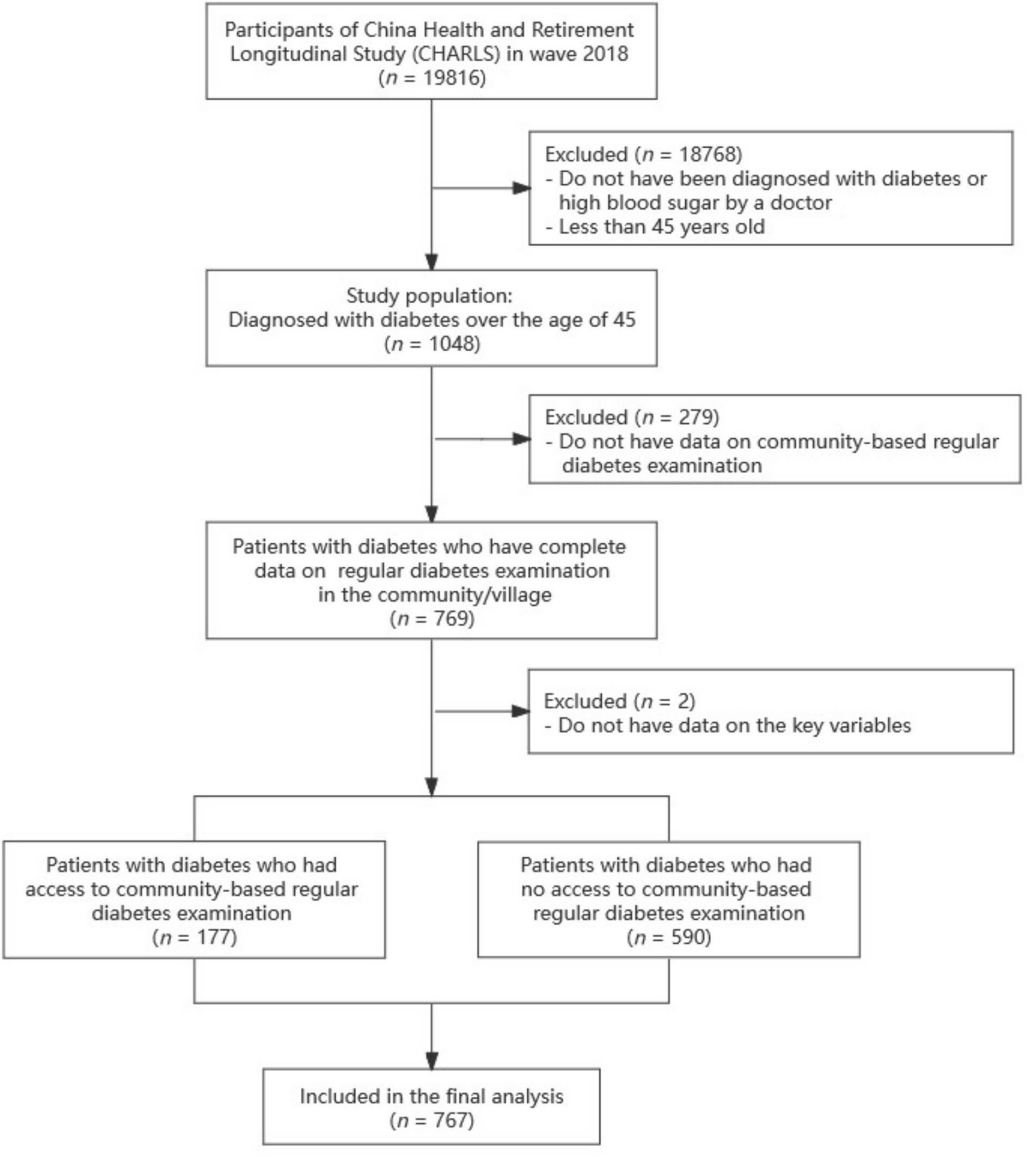
Process of sample inclusion.

The data used in this study were from the fourth wave of the China Health and Retirement Longitudinal Study (CHARLS), which is available for free at http://charls.pku.edu.cn. CHARLS is a large and representative national study hosted by Peking University's National School of Development. The baseline survey was conducted in 2011, followed by three subsequent waves in 2013, 2015, and 2018. The database covered the population from 450 communities/villages and 150 counties in 28 provinces of Mainland China. CHARLS data quality has been reported to be satisfactory in previous studies (25–27). Given the CHARLS database contains a wealth of information on individuals' SES, health status, and health service utilization, we employed it to investigate the inequalities of regular diabetes examination in the community and their relationship to healthcare utilization among patients with diabetes aged 45 years and older. As blood tests were not performed in the 2018 survey, diabetes was defined as self-reported diabetes diagnosed by a doctor (the questionnaire did not distinguish between type 1 and type 2 diabetes mellitus, so it was assumed that both types 1 and 2 were included). The procedure of sample inclusion is presented in Figure 1.

### Measures

#### Definition of healthcare utilization

We defined healthcare utilization in terms of outpatient and inpatient services utilization, which are dichotomous variables in the CHARLS data, by asking the respondents the single-choice questions of “In the last month, have you visited a public hospital, private hospital, public health center, clinic, or health worker's or doctor's practice, or been visited by a health worker or doctor for outpatient care? (Not including physical examination)” and “Have you received inpatient care in the past year?” The answers were categorized as yes and no.

#### Core explanatory variable

In CHARLS, diabetes examination included blood glucose test, urine glucose test, fundus examination, and micro-albuminuria test. The core explanatory variable of the study was the availability of community-based regular diabetes examination, which is measured by the question of “During the last year, have you had diabetes examination by community/village doctors regularly?” Responses to this question were dichotomized into yes or no.

#### Covariates

Based on the Anderson model, the potential predictors associated with healthcare utilization can be constructed in four aspects: individual characteristics, health behaviors, health outcome, and contextual factors. The individual characteristics included sex (male/female), age (45–60/61–75/>75), marital status (married and live with spouse/other), SES, and reimbursement rates of medical insurance. SES was assessed using two indicators of economic condition and education level. Per capital household expenditure is a more accurate reflection of economic condition and can help mitigate information bias than income, especially in low-income rural areas (28). In the CHARLS data, per capital household expenditure was obtained by dividing total household expenditure by the number of household member and then grouped into quantiles, with quantile 1 indicating the poorest and quantile 3 indicating the richest. The level of education was classified into three groups: uneducated, primary school or below, and secondary school or above. The classification of reimbursement rates of medical insurance into high, low, and no insurance was according to the fact that of the listed medical insurance, government medical insurance, urban employee medical insurance, and private medical insurance reimburse at a significantly higher rate than other insurances (29).

Drinking, frequency of social interaction (almost daily/almost every week/not regularly/never), and time for physical activities were used to assess health behaviors. Three categories of drinking were established: never, more than once a month, and less than once a month. Social interaction in this study mainly refers to some forms of group activities and informal interactions that are unique to Chinese older adults, such as square dancing, playing cards/mah-jongg, interacting with friends, etc. Social interaction has been shown to be associated with health status and health service utilization of older adults (16, 30). Greater healthcare utilization may be linked to less social interaction (31, 32). The frequency of social interaction was classified in this study as almost daily, almost every week, not regularly, and never. Time for physical activities was measured with a reference to the International Physical Activity Questionnaire (IPAQ), and the calculated weekly durations were divided into quantiles, with quantile 1 indicating the least and quantile 3 indicating the most (33, 34). The health outcome was measured by a self-rated health question that classified health status as good, fair or poor.

Contextual factors included health education, type of address, and region (eastern/middle/western). Health education is a dichotomous variable. If the respondents were advised by a physician to perform any of weight control, exercise, diet, smoking control, and foot self-care, they were considered to have received health education. Options for the type of address comprised city/town center, combination zone between urban and rural areas, village, and special zone. The division of regions and address types were defined based on the National Bureau of Statistics (35).

### Statistical analysis

#### Basic processing of data

We performed multiple imputation of five for observations with few missing values. Categorical data were described with frequency and percentage, and numerical data were described with mean and standard error (SE). The Stata 15.1 software was used for data analysis. Statistical significance was set at *p* < 0.05.

#### Concentration curve and concentration index

The concentration curve and concentration index (CI) were used to reflect inequalities in access to community-based regular diabetes examination by SES (economic condition and education level), type of address, and region. The CI is defined as twice the area between the concentration curve and the line of equity, and the concentration curve is obtained by plotting the cumulative percentage of the availability of community-based regular diabetes examination (*Y*-axis) against the cumulative percentage of the population ranked by economic condition quantiles, education level, type of address, and region (*X*-axis). The CI can be calculated using the following formula (36):


$$\begin{array}{l} CI=\;\frac{2}{\mu \times cov\left(h,r\right)} \end{array}$$

where *h* is the outcome of health event (the availability of community-based regular diabetes examination in this study), μ is the mean of h, and *r* is the fractional rank of individuals in the distribution used. The CI ranges between −1 and +1, and a value of zero denotes absolute fairness. If the CI takes a negative value, it means that the availability of regular diabetes examination in the community is more concentrated in the poor. Conversely, if the CI is positive, indicating community diabetes examination is more concentrated in the rich. Since the outcome variable in the study is binary, the bounds of CI do not vary between −1 and + 1. To correct this issue, we followed Wagstaff's suggestion (37), normalizing the CI by dividing estimated CI by 1 minus the mean (1–μ).

#### Propensity score matching

The other primary objective of this study was to estimate the impact of community-based regular diabetes examination on healthcare utilization among patients with diabetes. Given that the initial conditions of observed objects are heterogeneous, we applied propensity score matching (PSM) approach to reduce the potential bias caused by sample selection (38). The methodology of PSM is to make the observations as close as possible to the random experimental data through matching and resampling. According to the principles of PSM, the model for estimating the average treatment effect of the treated (ATT) was defined as:


$$\begin{array}{l} ATT=E\left(y_{1i}|D_{i}=1\right)-E\;\left(y_{0i}|D_{i}=1\right) \end{array}$$

where *D*_*i*_ is the treatment variable. *D*_*i*_ is 1 when survey respondents are in communities where regular diabetes examination is available (treatment group); otherwise, *D*_*i*_ is 0 (control group). *y*_*i*_ is the outcome variable of healthcare utilization among patients with diabetes, *y*_1*i*_ and *y*_0*i*_ respectively indicated the healthcare utilization among patients with diabetes in the treatment and control group. The 1:4 neighbor matching with replacement matching method was used with a caliper of 0.05 in the base case scenario. To verify the matching effect, we examined the achieved percentage of the bias reduction of each covariate after matching. Adequate matching refers to the bias should be <10%. Then, binary logit regression models were performed to compare the outcomes in the treatment group and their matched control group, before and after the application of sampling weights provided by CHARLS.

In order to check the robustness of the results, we used the nearest 1:1 neighbor matching with the caliper and kernel matching for sensitivity analysis. Regarding the nearest 1:1 neighbor matching, a caliper range of 0.05 was set. Kernel matching used a quadratic kernel, and the bandwidth was set to 0.06.

## Results

### Characteristics of the sample

A total of 767 patients with diabetes were included in the study. Before matching, 177 respondents (23.08%) were in the treatment group (with access to community-based diabetes examination) and 590 (76.92%) in the control group (without access to community-based diabetes examination). For the utilization of healthcare services, the respondents who visited a doctor and were admitted to a hospital accounted for 21.90 and 28.55%, respectively. After matching, we found 489 respondents consisting of 166 in the treatment group and 323 in the control group.

Table 1 outlines the descriptive information of the respondents before and after PSM. Before PSM, the respondents in the treatment and control groups were comparable in sex, marital status, education level, economic condition, frequency of social activities, time for physical activities, self-rated health, and region, but were significantly different in age, reimbursement rate of medical insurance, drinking, health education, and type of address (*p* < 0.05). After PSM, no significant differences were observed for these characteristics between the treatment group and the control group (*p* > 0.05).

**Table 1:** Characteristics of the sample.

**Variable**	**Before PSM**		**After PSM**	
	**Control group** **(*n* = 590)**	**Treatment group** **(*n* = 177)**	**Control group** **(*n* = 323)**	**Treatment group** **(*n* = 166)**
**Sex**				
Male	278 (47.12)	73 (41.24)	138 (42.72)	70 (42.54)
Female	312 (52.88)	104 (58.76)	185 (57.28)	96 (57.83)
**Age**				
45–60	268 (45.42)	58 (32.77)**	133 (41.18)	56 (33.73)
61–75	275 (46.61)	101 (57.06)	169 (52.32)	96 (57.83)
>75	47 (7.97)	18 (10.17)	21 (6.50)	14 (8.43)
**Marital status**				
Married and lived with spouse	459 (77.80)	147 (83.05)	257 (79.57)	138 (83.13)
Other	131 (22.20)	30 (16.95)	66 (20.43)	28 (16.87)
**Education level**				
Uneducated	120 (20.34)	50 (28.25)	70 (21.67)	43 (25.90)
Primary school or below	246 (41.69)	69 (39.98)	151 (46.75)	67 (40.36)
Secondary school or above	224 (37.97)	58 (32.77)	102 (31.58)	56 (33.73)
**Economic condition (quantile)**				
Q1 (poorest)	154 (26.10)	61 (34.46)	97 (30.03)	56 (33.73)
Q2	198 (33.56)	65 (36.72)	120	59 (35.54)
Q3 (richest)	238 (40.34)	51 (28.81)	106 (32.82)	51 (30.72)
**Reimbursement rate of medical insurance**				
High	158 (26.78)	25 (14.12)**	63 (19.50)	25 (15.06)
Low	423 (71.69)	150 (84.75)	255 (78.95)	139 (83.73)
No insurance	9 (1.53)	2 (1.13)	5 (1.55)	2 (1.20)
**Drinking**				
Never	401 (67.97)	137 (77.40)**	237 (73.37)	129 (77.71)
More than once a month	47 (7.97)	16 (9.04)	23 (7.12)	16 (9.64)
Less than once a month	142 (24.07)	24 (13.56)	63 (19.50)	21 (12.65)
**Frequency of social activities**				
Almost daily	205 (34.75)	55 (31.07)	108 (33.44)	53 (31.93)
Almost every week	70 (11.86)	15 (8.47)	35 (10.84)	15 (9.04)
Not regularly	82 (13.90)	26 (14.69)	49 (15.17)	24 (14.46)
Never	233 (39.49)	81 (45.76)	131 (40.56)	74 (44.58)
**Time for physical activities**				
Q1 (least)	205 (34.75)	51 (28.81)	93 (28.79)	47 (28.31)
Q2	198 (33.56)	57 (32.20)	108 (33.44)	54 (32.63)
Q3 (most)	187 (31.69)	69 (38.98)	122 (37.77)	65 (39.16)
**Health education**				
Yes	409 (69.32)	154 (87.01)***	264 (81.73)	146 (87.95)
No	181 (30.68)	23 (12.99)	59 (18.27)	20 (12.05)
**Type of address**				
The center of city/town	157 (26.61)	30 (16.95)**	67 (20.74)	28 (16.87)
Combination zone between urban and rural areas	70 (11.86)	13 (7.34)	30(9.29)	11(6.63)
Village	357 (60.51)	134 (75.71)	224 (69.35)	127 (76.51)
Special area	6 (1.02)	—	2(0.65)	0(0.00)
**Region**				
Eastern	228 (38.64)	75 (42.37)	120 (37.15)	72 (43.37)
Middle	183 (31.02)	50 (28.25)	103(31.89)	43(25.90)
Western	179 (30.34)	52 (29.38)	100(30.96)	51(30.72)
**Self-rated health**				
Good	77 (14.18)	23 (13.77)	35 (10.84)	23 (13.86)
Fair	263 (48.43)	68 (40.72)	152 (47.06)	58 (40.96)
Poor	203 (37.38)	76 (45.51)	136 (42.11)	75 (45.18)

Percentages are in parentheses. Significance: 5% (**) and 1% (***) for χ^2^ tests for each treatment group (with access to community-based diabetes examination) vs. the control group (without access to community-based diabetes examination).

### Inequalities in community-based diabetes examination

**Figure 2:**
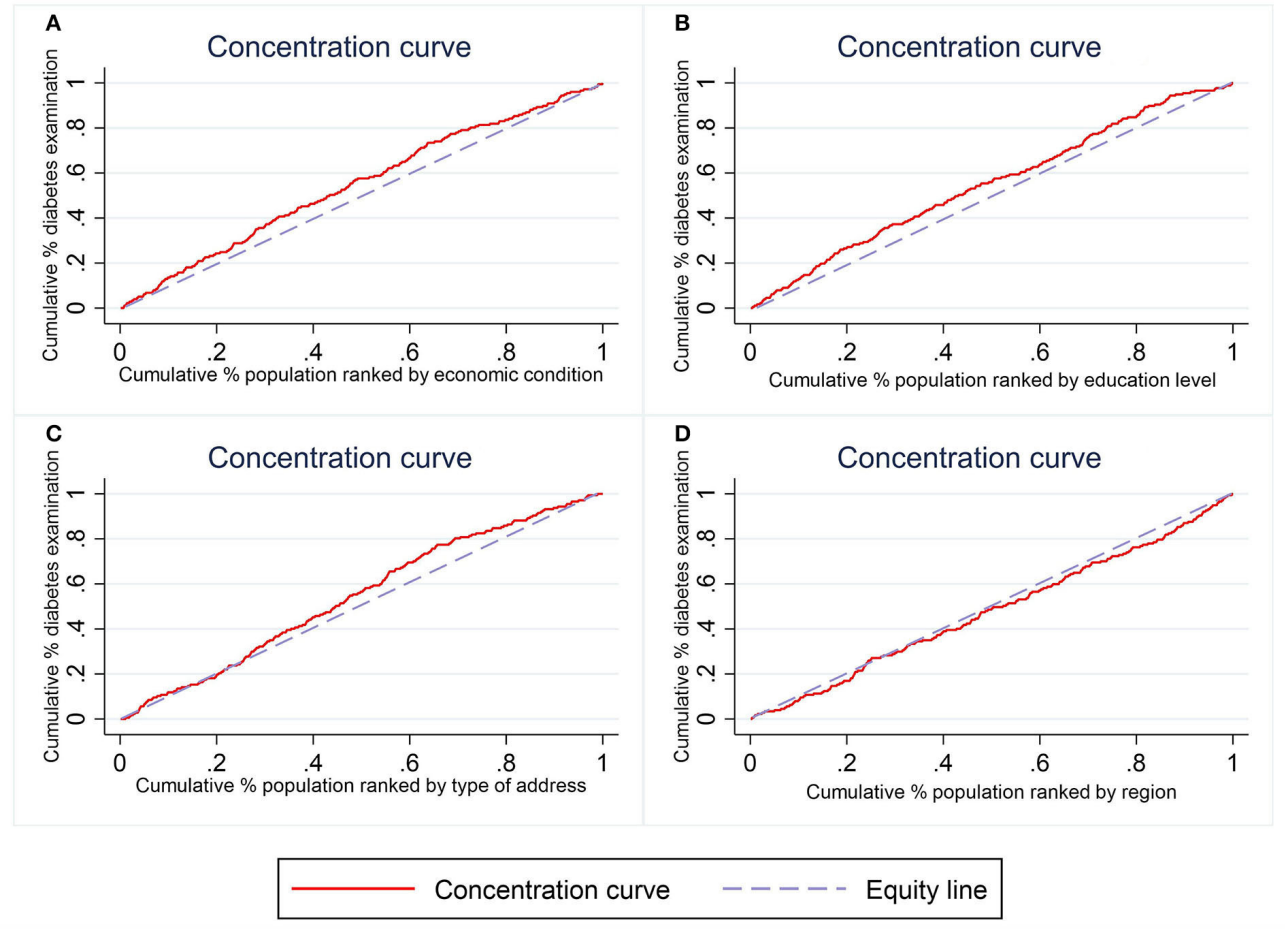
Concentration curves of inequalities in community-based diabetes examination.

Figure 2 plots the concentration curves of inequalities on community-based diabetes examination for the four variable of interest, i.e., economic condition, education level, type of address, and region, with CIs approximately equal to −0.104 (*p* = 0.0035), −0.092 (*p* = 0.0129), −0.103 (*p* = 0.0007), and 0.025 (*p* = 0.4971), respectively. The relative position of the concentration curve and the equity line identifies the magnitude of inequalities. The concentration curves for economic condition, education level, and region were above their respective equity lines, indicating that community-based diabetes examinations were more concentrated among people in poor economic circumstances, with lower education levels, and in less developed areas. This suggests that there is inequality in the distribution of community-based diabetes examination, and the inequality favoring those who are poorer, less educated, and live in undeveloped areas. Although slightly below the equity line, the curve for geographical regions has a 95% confidence interval that contains 0, which indicates that community-based diabetes examinations were not statistically different across eastern, middle, and western regions.

**Figure 3:**
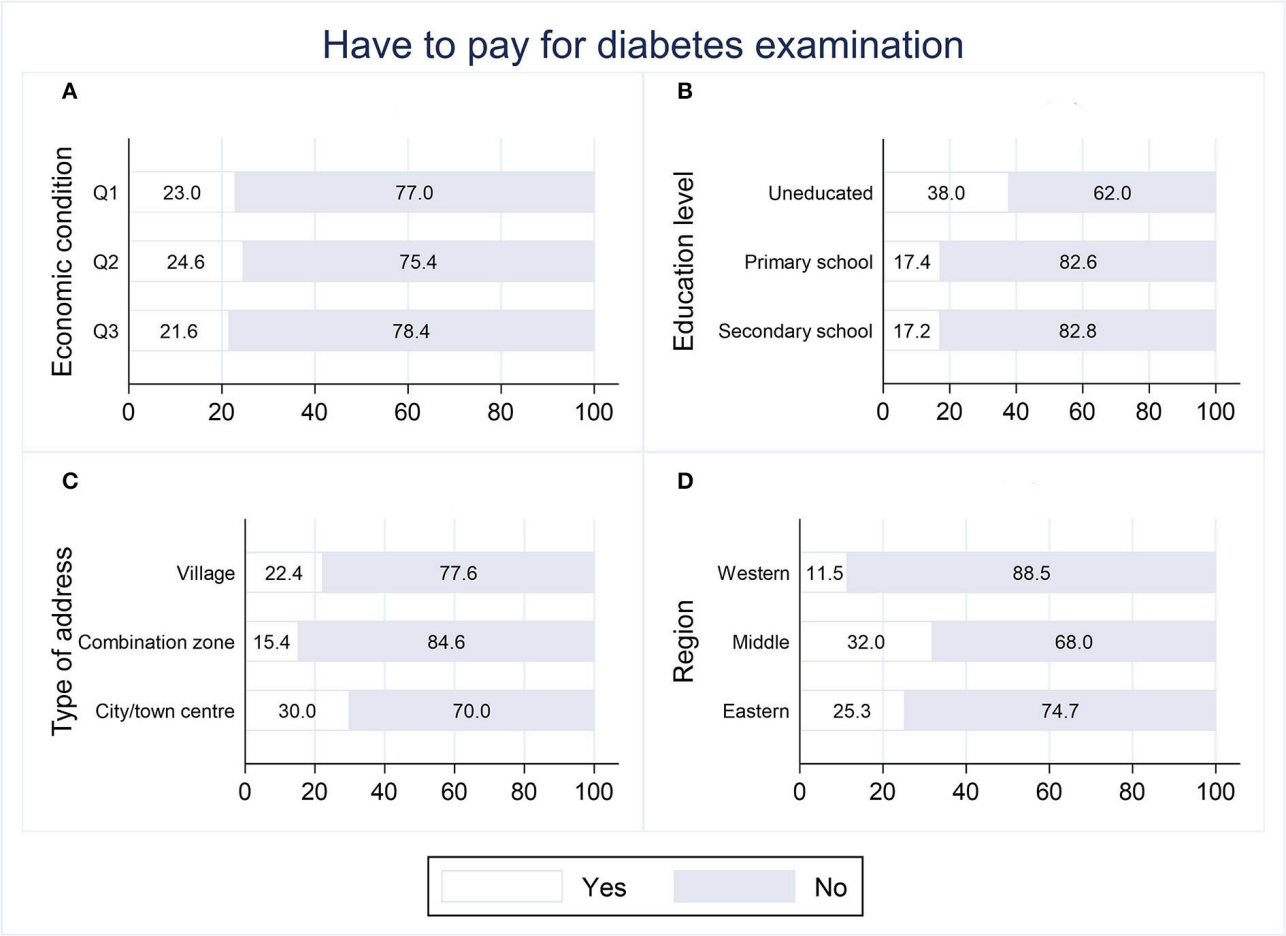
Distribution of whether have to pay for community-based diabetes examination.

**Figure 4:**
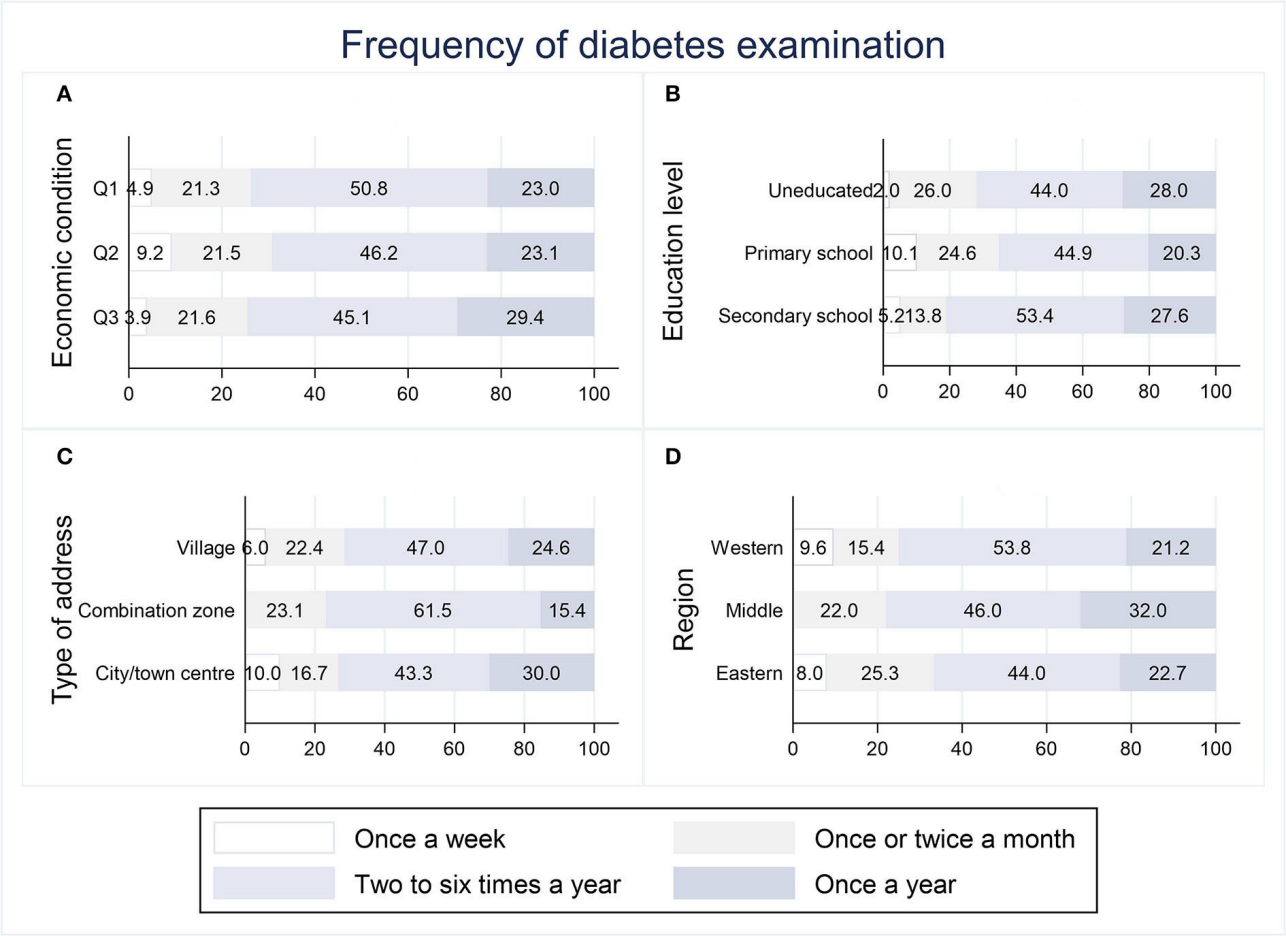
Distribution of frequency of community-based diabetes examination.

Figures 3, 4 show the distribution of socioeconomically relevant variables at both the individual and regional levels for whether payment was required and the frequency of examination in the 177 samples from the treatment group. The proportion of those who did not have to pay for diabetes examinations was overwhelmingly dominant in all four variables, with a range of 62.0–88.5%. Among them, the educated and people in the western region had a higher percentage of not having to pay for diabetes examinations compared to the uneducated and people in the eastern and middle regions (*p* < 0.05). The frequency of diabetes examination was roughly identical across groups in terms of economic condition, education level, type of address, and region. The overall picture was that the highest frequency was two to six times a year (43.3–61.5%), followed by once a year (15.4–32.0%), once or twice a month (13.8–26.0%), and once a week (0–10.1%).

### Propensity score matching analysis

**Figure 5:**
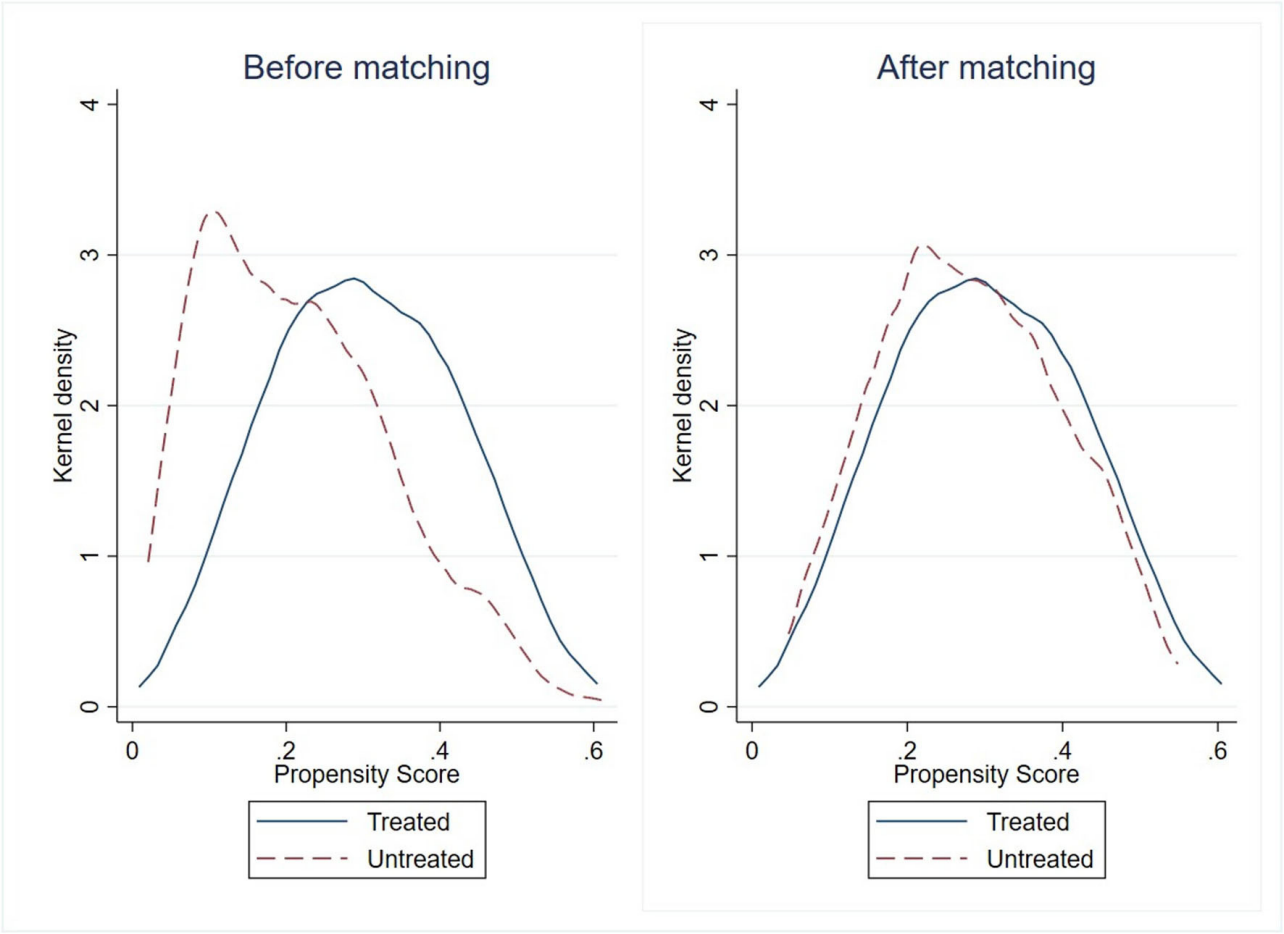
Kernel density estimates.

**Figure 6:**
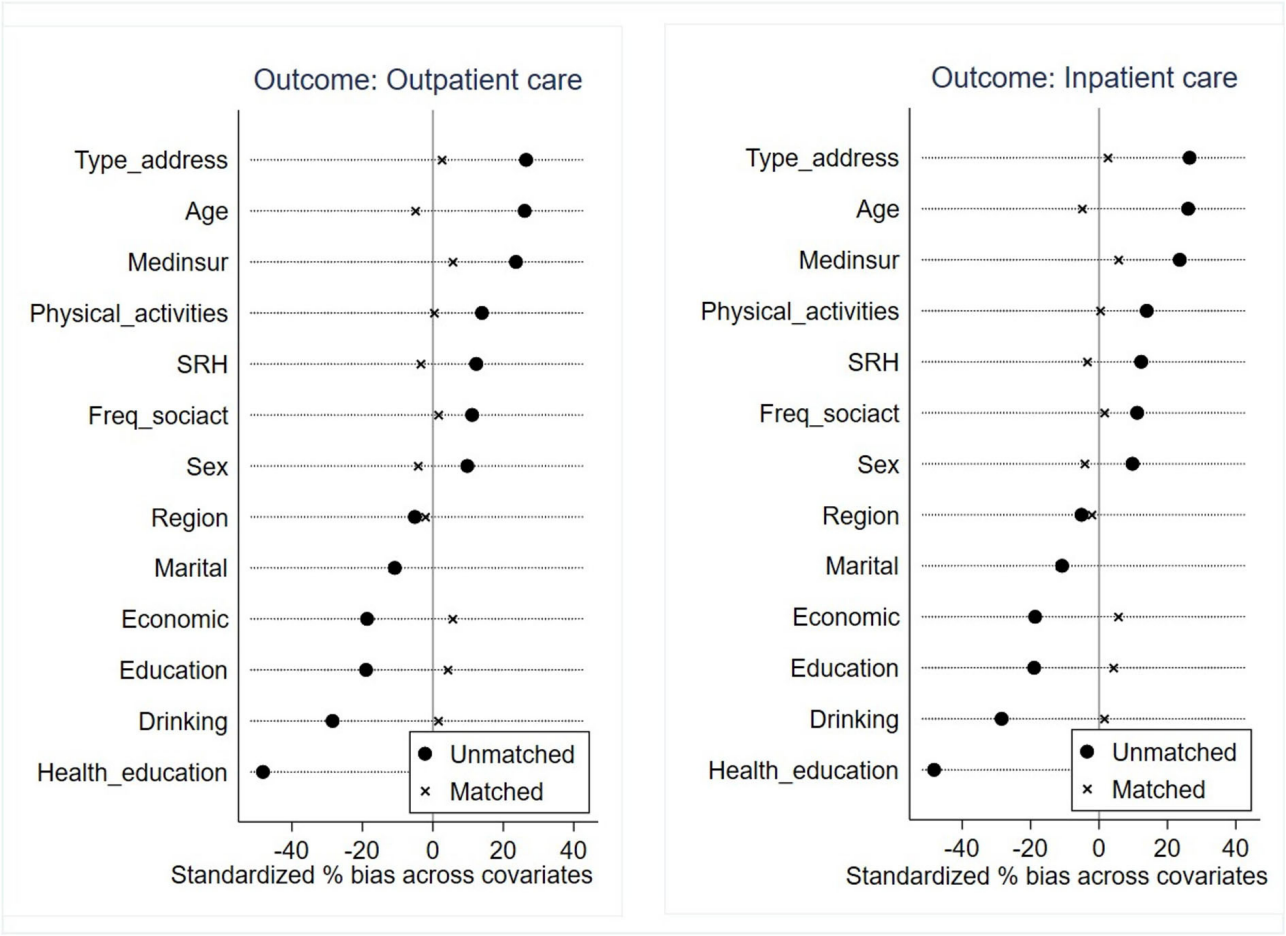
Balance test of the covariates.

As illustrated in Figure 5, the propensity scores for the treatment and control groups overlapped sufficiently in distribution after matching. This suggested that it was likely to find an appropriate match for most control group respondents. Furthermore, according to the results of the balance test, the standardized differences (% bias) after matching were considerably reduced and were all within the 10% threshold (Figure 6). Consequently, the common support condition and matching effect were satisfied.

The unweighted logit analysis of the matched samples showed that the respondents with access to community-based diabetes examination not only had a higher probability of outpatient care utilization (OR = 1.989, 95%CI: 1.156–3.974) but were also less likely to utilize inpatient care (OR = 0.544, 95%CI: 0.325–0.909). After the application of sampling weights, the respondents with access to community-based diabetes examination not only had a higher probability of outpatient care utilization (OR = 2.162, 95%CI: 1.138–4.107) but were also less likely to utilize inpatient care (OR = 0.569, 95%CI: 0.313–1.035) (Table 2).

**Table 2:** Effects of community-based diabetes examination on healthcare utilization (logit model).

**Healthcare utilization**	**Unweighted**			**Weighted**		
	**OR^a^**	**SE**	**95%CI**	**OR^a^**	**SE**	**95%CI**
Outpatient care utilization	1.989**	0.551	1.156–3.974	2.162**	0.708	1.138–4.107
Inpatient care utilization	0.544**	0.143	0.325–0.909	0.569*	0.174	0.313–1.035

Significance: 10% (*) and 5% (**); ^**a**^Covariates including sex, age, marital status, education level, economic condition, reimbursement rate of medical insurance, drinking, frequency of social activities, time for physical activities, health education, self-rated health, type of address, and region were adjusted for calculation.

Tables 3–5 present the results of sensitivity analyses. Both logit models and ATT estimations indicated that the increased probability of outpatient care utilization and decreased probability of inpatient care utilization were associated with community-based diabetes examination, no matter which matching method was used. Additionally, we combined the data from CHARLS 2015 and 2018 into a single data pool without distinguishing between years. The weighted analysis demonstrated the robustness of the results.

**Table 3:** Sensitivity analyses for the effects of community-based diabetes examination on healthcare utilization.

**Healthcare utilization**	**1:1 matching (128:122)**			**Kernel matching (137:134)**		
	**OR^a^**	**SE**	**95%CI**	**OR^a^**	**SE**	**95%CI**
Outpatient care utilization	1.483*	0.353	0.929–2.366	1.581*	0.499	0.851–2.935
Inpatient care utilization	0.443**	0.144	0.235–0.838	0.762*	0.115	0.567–1.025

Significance: 10% (*) and 5% (**); ^**a**^Covariates including sex, age, marital status, education level, economic condition, reimbursement rate of medical insurance, drinking, frequency of social activities, time for physical activities, health education, self-rated health, type of address, and region were adjusted for calculation.

**Table 4:** Average treatment effect (ATT) on the treated (with access to community-based diabetes examination).

**Method**	**Outpatient care utilization**		**Inpatient care utilization**	
	**ATT**	**SE**	**ATT**	**SE**
1:1 matching	0.251**	0.051	0.246**	0.059
Kernel matching	0.251**	0.043	0.246**	0.051

Significance: 5% (**).

**Table 5:** Combined data for CHARLS 2015 and 2018: Effects of community-based diabetes examination on healthcare utilization (logit model).

**Healthcare utilization**	**Unweighted**			**Weighted**		
	**OR^a^**	**SE**	**95%CI**	**OR^a^**	**SE**	**95%CI**
Outpatient care utilization	1.260**	1.119	1.047–1.517	1.289**	1.136	1.049–1.584
Inpatient care utilization	0.849	0.088	0.694–1.039	0.801*	0.096	0.633–1.013

Significance: 10% (*) and 5% (**); ^**a**^Covariates including sex, age, marital status, education level, economic condition, reimbursement rate of medical insurance, drinking, frequency of social activities, time for physical activities, health education, self-rated health, type of address, and region were adjusted for calculation.

## Discussion

To the best of our knowledge, this is the first study to examine the status and inequalities of community-based regular diabetes examination and its effect on the likelihood of healthcare utilization among patients with diabetes. The findings suggest that community-based regular diabetes examination is currently at a low level in China, and there are distributional inequalities across populations who are favorable to the socioeconomically disadvantaged. The PSM analysis confirmed that community-based regular diabetes examination was associated with increased outpatient care utilization and decreased inpatient care utilization.

Beyond our expectations, unlike most studies in which health resources are concentrated in people with advantageous SES (39–41), community-based diabetes examinations in this study were more concentrated among the disadvantaged (with worse economic condition and lower education level, and live in less developed areas). Possible reasons for this can be attributed to the fact that low-socioeconomic groups are usually more concerned with less expensive or free public services and are associated with more frequent visits to primary healthcare services, whereas patients with a higher SES visit doctors more frequently and may be unaware of the availability of services such as diabetes examination in their communities (42, 43). Additionally, significant central government transfers are made to less developed areas, such as rural areas. Rural populations face greater barriers to healthcare services than urban populations, and rural diabetes patients perform worse in terms of diagnosis and control of the disease (19, 27). A hopeful and optimistic hypothesis is that government investment in rural primary diabetes examination will help alleviate this problem.

Despite such a distribution favors vulnerable populations, there is still an inherent health inequality. In terms of diabetes examination itself, health equity does not compensate for the poor by the denial of health rights to the rich. That is to say, people with higher SES are equally entitled to demand community-provided diabetes services. On the other hand, from the perspectives of healthcare delivery equity and efficient allocation of medical resources, the results indicated that community-based diabetes examination is less available to the wealthy (home care by themselves or access to tertiary care, which we do not know based on this dataset), which excludes them from being targeted for community-provided services, regardless of whether they would utilize those services or not. Regular diabetes examinations at the community level are sufficient for daily diabetes management, and increasing the availability of community-based diabetes examination among the rich could be a way to reduce occupation of specialized healthcare in routine chronic disease management.

In addition to being unevenly distributed across populations, the coverage of community-based diabetes examination was relatively narrow, with results showing that less than a quarter (23.08%) of respondents had access to it, and a portion of them had to pay for it. For the older generation in China, schooling as a child or adolescent is not a common option that most people can afford, making educational attainment an important indicator of social stratification (44). The uneducated generally reside in less socioeconomically advantaged communities, which often pose barriers to health resources and services (45). In the case of this study, communities with a high proportion of uneducated residents were more likely to charge for diabetes examinations that were offered. As a result of its less developed economy, the western region has long received financial subsidies from the state and has a greater share of government healthcare expenditures. According to data on special transfer payments disclosed by the Ministry of Finance and the National Health Commission in 2021 (46), per capita subsidies for basic public healthcare in the western region were 2.15 and 1.15 times higher, respectively, than in the eastern and middle regions, and per capita government health expenditures were 1.10 and 1.27 times higher. This suggests that individuals in the west are more likely to be exempt from having to pay for community-based diabetes examination. In terms of frequency, the frequency that accounted for the highest percentage was 2–6 times per year, which is adequate for fundus examinations, micro-albuminuria tests, etc., but still far from sufficient for blood glucose test. Overall, the results indicate that community-based diabetes examination is currently at a low level of limited coverage and low frequency.

After controlling for confounding variables, PSM results suggested that community-based diabetes examination has a significant positive impact on outpatient care utilization among people with diabetes. While this result appears to contradict the original goal of “keeping chronic disease management in the community,” it should also be noted that the findings show the potential of community-based diabetes examination to release health demands of person with diabetes. Some people avoid seeking healthcare due to stigma or time, financial, transportation, and knowledge constraints (47), but avoiding healthcare increases the disease's preventable risks for people with diabetes (23), resulting in a vicious cycle. Regular community-based diabetes examination can act as a booster for diabetes management. Of course, healthcare providers are unable to address multiple health concerns and provide all necessary medical advices during a single time-limited diabetes examination appointment (23), but increased utilization of outpatient services may help remedy this gap in the current context of low examination frequency. Previous studies have found that regular health visits can ameliorate fear of disease examination and treatment, improve medical trust and self-efficacy, and increase health awareness for self-management and active treatment (48, 49). The study reaffirms this point of view. Additionally, community-based diabetes examination was found to have a negative and significant effect on inpatient care utilization. One plausible explanation is that outpatient care does not represent the severity of disease as inpatient visits do. Diabetes is a chronic condition that necessitates long-term management, and patients with diabetes who are properly managed in the community and by themselves do not require hospitalization unless they have other conditions unrelated to diabetes.

This study also adds to the literature by addressing endogeneity in the diabetes examination–healthcare utilization link. We employed the PSM approach to correct for the sample selection bias and potential structural confounding. This is achieved by performing analyses between participants who are exchangeable between those with and without access to community-based diabetes examinations, on the basis of a set of predictors derived from the Andersen model. In doing this, observed differences in the outcomes (specifically, outpatient and inpatient care utilization) between the treatment and control groups are inferred to be the result of the treatment (community-based diabetes examination) alone (50). Our results show that the associations between community-based diabetes examination and healthcare utilization are robust net of the selection bias.

The extrapolation of the present study needs to be considered in conjunction with previous CHARLS studies. Previous CHARLS studies have shown, first, that self-reported rates of diabetes ranged between 5 and 7%, tending to underestimate the true prevalence of diabetes (51, 52). The lack of biomedical data in this study, the percentage of self-reported diabetes was considered to be the overall prevalence of diabetes and thus failed to derive diabetes awareness, which is important for engagement in community-based diabetes examination. Second, middle-aged and older adults who are younger and have a lower SES are more likely to be unaware that they have diabetes, due to neglect of their health and poor accessibility to health resources for medical examination. The less developed the region, the lower the level of agreement between the prevalence of diabetes and self-reported measurements of diabetes (52). The eastern region of this study had a higher proportion of the sample than the central and western regions, and the proximity of self-reports to diabetes prevalence varies across regions, whereas self-reports were associated with responses to community-based diabetes examination (CHARLS questionnaire procedure was set up such that data on community diabetes examination could only be collected from people who self-reported having diabetes), which could potentially influence the results of health inequalities in community-based diabetes examination. Third, some respondents who had diabetes but were unaware of the condition were excluded. It is possible that some respondents lived in communities where regular diabetes examinations were available at the time of interview but were unaware of them because many respondents are socioeconomically disadvantaged and face structural barriers to seeking health information. As a result, the actual coverage of community-based diabetes examinations may be underestimated. Furthermore, the limited dataset does not allow us to conduct additional analyses; with a small sample size on the one hand, some key variables were missing, such as smoking, and a health behavior variable was not included in this study; on the other hand with all selected variables from the CHALRS 2018 questionnaire, we were unable to analyze what was of added-value to the study but beyond the questionnaire. In addition, the lack of blood test data leads to an underestimation of diabetes prevalence. The percentage of self-reported diabetes is considered to be the total prevalence of diabetes and thus failing to derive diabetes awareness, which is important for engagement in community-based diabetes examination.

## Conclusion

In conclusion, this study sheds new light on the inequalities of community-based regular diabetes examination and its impact on healthcare utilization in individuals with diabetes. We present new evidence of health inequalities that favor patients with low SES. Diabetes risk is increasing in China for both high- and low-SES individuals, but underserved patients of low SES are in greater need of additional support from the health system. Aside from that, overall health equity and resource allocation could be further optimized. At the same time, we observed that diabetes examination had a positive effect on the demand for outpatient visits. The message of our study is that expanding the coverage and depth of community-based regular diabetes examination should be considered by policymakers in public health and in other health policy priorities to strengthen disease control and management of diabetes and prediabetes, assist them in confronting their health needs, and promote health.

## Data availability statement

The raw data supporting the conclusions of this article will be made available by the authors, without undue reservation.

## Ethics statement

The studies involving human participants were reviewed and approved by Research Ethics Committees of Peking University. The patients/participants provided their written informed consent to participate in this study.

## Author contributions

YC and QD contributed to the conception and design of the study. QD conducted the data reduction, statistical analysis, and drafted the manuscript. QD, YW, and YC contributed to the data interpretation and reviewed the manuscript. All authors have approved the manuscript before submission.

## Conflict of interest

The authors declare that the research was conducted in the absence of any commercial or financial relationships that could be construed as a potential conflict of interest.

## Publisher's note

All claims expressed in this article are solely those of the authors and do not necessarily represent those of their affiliated organizations, or those of the publisher, the editors and the reviewers. Any product that may be evaluated in this article, or claim that may be made by its manufacturer, is not guaranteed or endorsed by the publisher.
